# Proteome and lysine acetylome analysis reveals insights into the molecular mechanism of seed germination in wheat

**DOI:** 10.1038/s41598-020-70230-8

**Published:** 2020-08-10

**Authors:** Weiwei Guo, Liping Han, Ximei Li, Huifang Wang, Ping Mu, Qi Lin, Qingchang Liu, Yumei Zhang

**Affiliations:** 1 Shandong Provincial Key Laboratory of Dryland Farming Technology/College of Agronomy, Qingdao Agricultural University, Qingdao Shandong, 266109 China; 2 Laboratory of Crop Heterosis and Utilization, Ministry of Education, China Agricultural University, Beijing, 100193 China

**Keywords:** Proteomic analysis, Plant morphogenesis

## Abstract

Seed germination is the first stage in wheat growth and development, directly affecting grain yield and quality. As an important post-translation modification, lysine acetylation participates in diverse biological functions. However, little is known regarding the quantitative acetylproteome characterization during wheat seed germination. In this study, we generated the first comparative proteomes and lysine acetylomes during wheat seed germination. In total, 5,639 proteins and 1,301 acetylated sites on 722 proteins were identified at 0, 12 and 24 h after imbibitions. Several particularly preferred amino acids were found near acetylation sites, including K^ac^S, K^ac^T, K^ac^K, K^ac^R, K^ac^H, K^ac^F, K^ac^N, K^ac^*E, FK^ac^ and K^ac^*D, in the embryos during seed germination. Among them, K^ac^H, K^ac^F, FK^ac^ and K^ac^K were conserved in wheat. Biosynthetic process, transcriptional regulation, ribosome and proteasome pathway related proteins were significantly enriched in both differentially expressed proteins and differentially acetylated proteins through Gene Ontology and Kyoto Encyclopedia of Genes and Genomes enrichment analysis. We also revealed that histone acetylation was differentially involved in epigenetic regulation during seed germination. Meanwhile, abscisic acid and stress related proteins were found with acetylation changes. In addition, we focused on 8 enzymes involved in carbohydrate metabolism, and found they were differentially acetylated during seed germination. Finally, a putative metabolic pathway was proposed to dissect the roles of protein acetylation during wheat seed germination. These results not only demonstrate that lysine acetylation may play key roles in seed germination of wheat but also reveal insights into the molecular mechanism of seed germination in this crop.

## Introduction

Wheat (*Triticum aestivum* L.), which is also known as bread wheat, is one of the most important cereal crops in the world. Given its sessile nature, wheat is constantly exposed to a changing environment and must adapt its endogenous status to these changes rapidly to ensure survival. Protein posttranslational modifications (PTMs), which play important roles in many kinds of biological processes, can help to trigger a more rapid response^1^.

PTMs can change protein functions by introducing new functional groups, such as acetyl, phospho, ubiquityl, methyl, succinyl and crotonyl groups^2^. Among them, lysine acetylation, including non-nuclear protein and histone acetylation, is an evolutionarily conserved PTM that occurs in both prokaryotic and eukaryotic organisms^3^. Histone acetylation is a leading epigenetic mechanism, and its role has been extensively investigated in regulating gene transcription^4^. In addition to histones, non-histone acetylation has also been found in many cellular compartments and regulates a wide variety of important cellular processes, such as enzymatic activity, cell morphology, protein stability, protein interactions and metabolic pathways^4^.

Seed germination represents the developmental transition from maturation drying to a sustained metabolic rate in preparation for seedling establishment. Germination, which is strictly controlled by endogenous and environmental signals, is also considered to be the first growth stage in the plant life cycle^5^. Lysine acetylation has been reported to participate in diverse biological process and events in various plant species through acetylproteome characterization analysis^1,6^. In the latest researches, it was reported that lysine acetylation involved in fungal infection response, meiosis and tapetum function and diurnal cycle in plant^7–9^. As to seed germination process, the first growth stage in the plant life cycle, rice and *Picea asperata* have been studied and there are 699 acetylated sites in 389 proteins in rice seed embryo and 1,079 acetylation sites in 556 proteins in *Picea asperata* somatic embryos during germination stage^10,11^. Thus the potential underlying mechanisms of protein acetylation regulating seed germination still requires further exploration.

Compared with qualitative analysis, quantitative analysis can reveal the dynamic protein expression profile changes and global protein acetylation level alteration at different development stages. Wang et al. performed the quantitative acetylome study at early seed development stage in rice and identified 370 differentially acetylated peptides in 268 acetylation proteins; these differentially acetylated proteins participated in multiple metabolisms in rice seed early development after pollination^12^. Zhu et al. conducted the quantitative acetylproteome analysis in developing wheat grains following flowering stages under water deficit condition and found the proteins with changed acetylation level involved in diverse metabolic pathways and had important regulating roles in wheat starch biosynthesis, grain development and yield formation^13^. The lysine influenced seed development and maturation has also been studied in soybean^14^.

Nonetheless, compared with seed development and maturation stage, the quantitative acetylome analysis at seed germination stage hasn’t been study. In this study, we carried out the quantitative acetylome analysis in wheat seed embryos of 0, 12 and 24 h after imbibitions (HAI). The quantitative proteome analysis was also performed with the purpose of providing more information about metabolism regulation in germinating seeds. Our study may serve as an important resource for structure and functional characterization of lysine acetylation in seed germination, which is of significance in both plant biology and agriculture science.

## Result and discussion

### Experiment design and workflow

**Figure 1:**
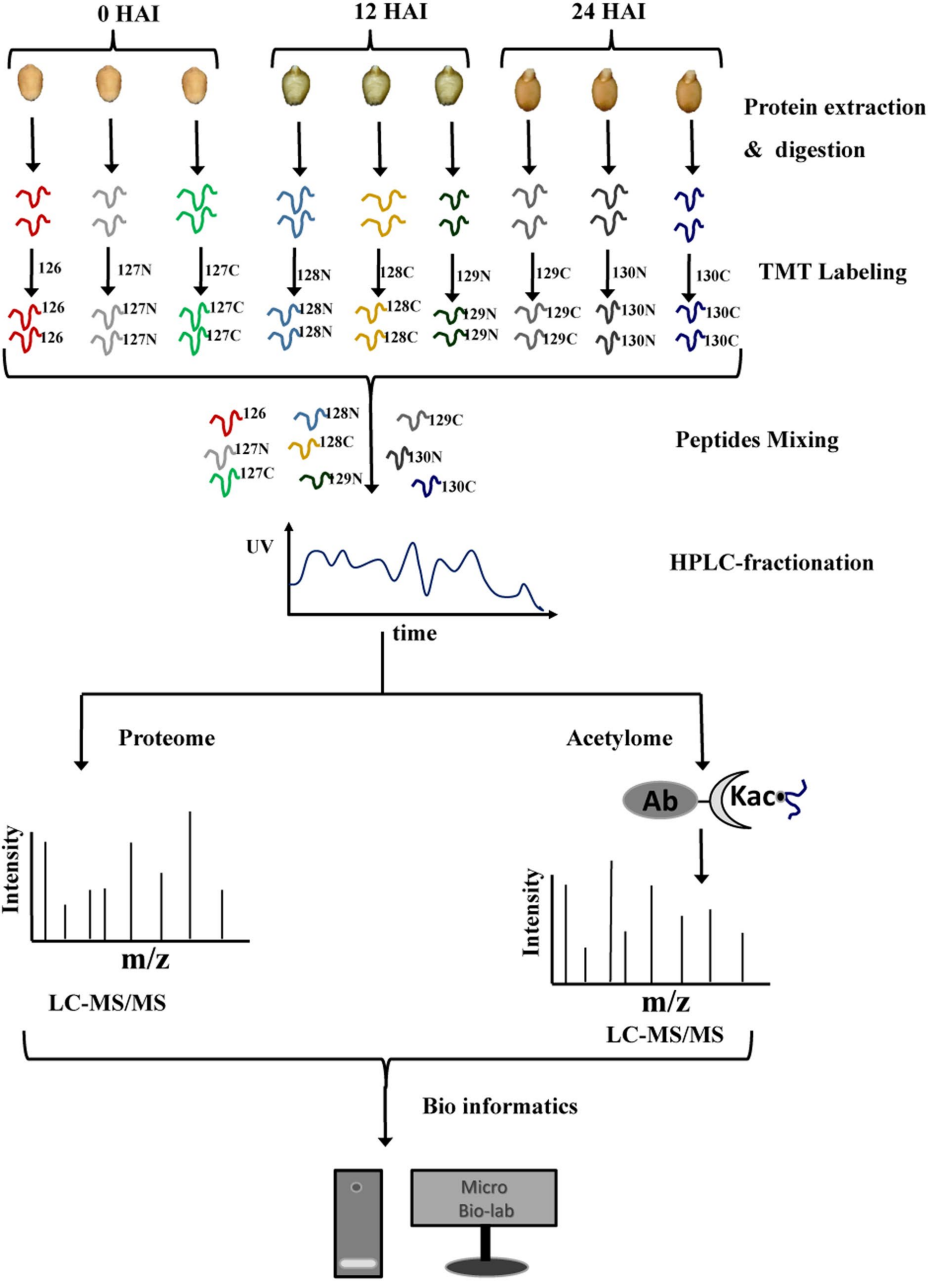
The workflow for the proteome and acetylome analysis of wheat seed embryo. HAI, hour after imbibitions; TMT, tandem mass tag; HPLC, high performance liquid chromatography; LC–MS/MS, liquid chromatography-tandem mass spectrometry.

The purpose of this study is to uncover the mechanisms regulating wheat seed germination at global proteome level and acetylome level. Firstly, seed morphological features observation and germination rates measurement at 0, 4, 8, 12, 16, 24 and 32 h after imbibitions (HAI) were performed. Based on the physiological index results, the seed embryos of 0 HAI, 12 HAI and 24 HAI were selected for the quantitative proteome and quantitative acetylome analysis. Three independent biological replications of each stage were performed. The global workflow for the proteome and acetylome analysis was shown in Fig. 1, mainly including protein extraction and trypsin digestion, Tandem Mass Tag (TMT) labelling and mixing, HPLC fractionation, acetylated peptides enrichment (only for acetylome analysis), LC–MS/MS acquisition and bioinformatics analysis.

### Seed morphologic observation and germination rate calculation

**Figure 2:**
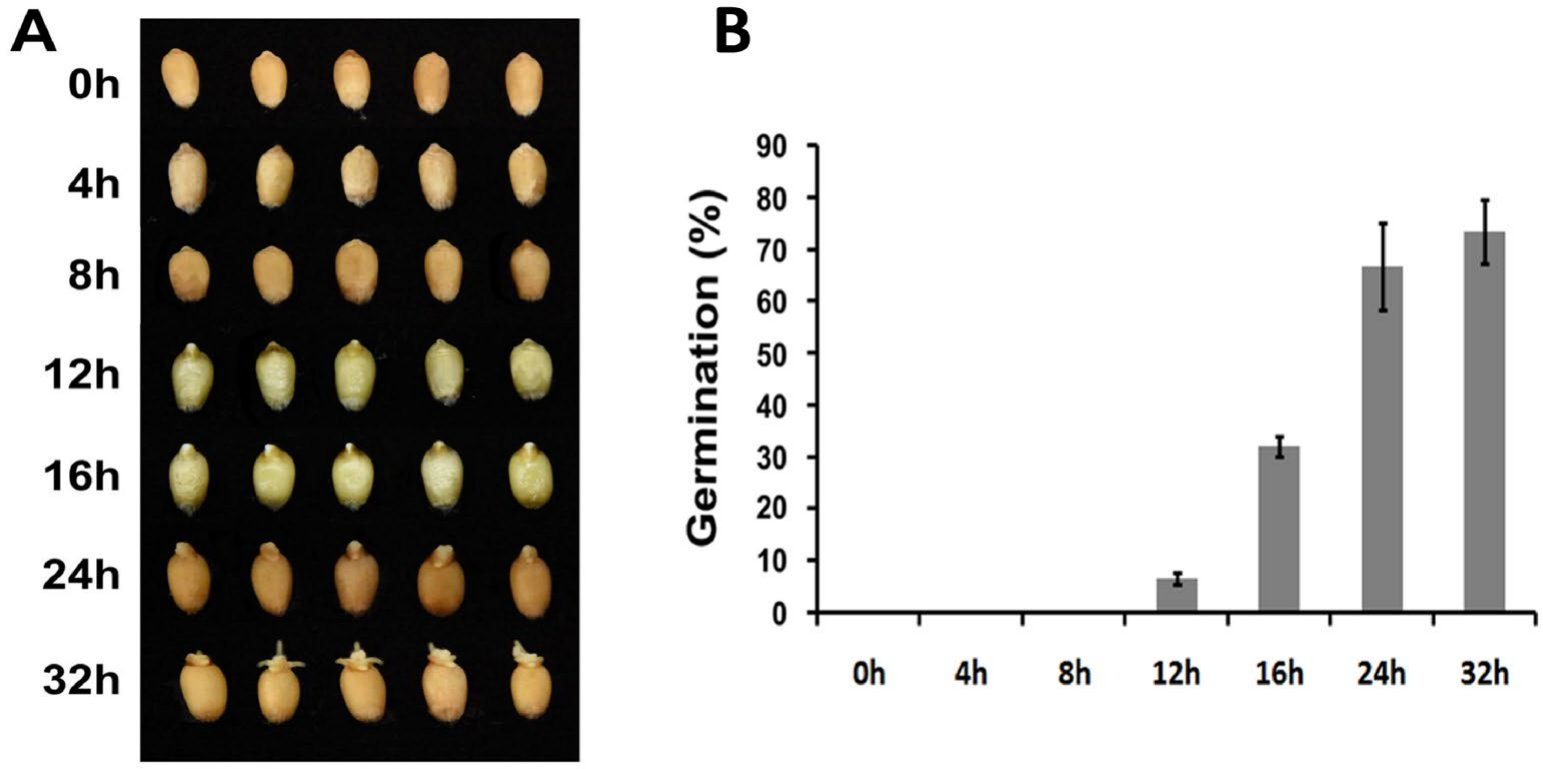
Seed germination of QM6. (**A**) Seed morphological changes during germination. (**B**) Germination time course of QM6.

As shown in Fig. 2A, seed size increased gradually with water imbibition. Radicals began to break through the episperm at the stage of 12 HAI, which could be regarded as the relatively early stage of seed germination. With imbibition time increase, more and bigger radicals were observed. At 24 HAI, significantly inflated radicals were observed and three radicles were visible at 32 HAI. A time-course calculation of seed germination rate was calculated with the standard radical breaking through the episperm is regarded as germination (Fig. 2B). At 0 HAI, 4 HAI and 8 HAI, no visible radicals were observed and the germination rate was calculated as zero. At 12 HAI, the germination rate was only 6.7% and increased dramatically at the following time intervals. The germination rate reached 67% at 24 HAI while only slight germination rate increase was observed at 32 HAI with 73%. The 24 HAI and 32 HAI could be regarded as the relatively later stages of seed germination. Based on the seed morphological features observation and germination rates calculation result, 0 HAI, 12 HAI (early stage of germination) and 24 HAI (later stage of germination) were selected for the proteome and acetylome analysis.

### Profile of proteome in embryos of wheat at different HAI intervals

In total, 6,927 protein groups were identified from wheat seed embryos, and 5,639 proteins were quantified (Supplementary Table S1). With the threshold changed protein expression intensity > twofold (which was the highest threshold during protein omics analysis) and *P* < 0.05, the differentially expressed proteins (DEPs) were screened among three selected HAI intervals (Supplementary Table S2). Compared with 0 HAI, 12 HAI induced 960 DEPs (435 up-regulated and 525 down-regulated) and 24 HAI resulted in 1,428 DEPs (693 up-regulated and 735 down-regulated). Relatively less DEPs (129 up-regulated and 85 down-regulated) were identified between 12 HAI vs 24 HAI.

### Enrichment-based cluster analysis of the DEPs

#### GO enrichment based cluster analysis

**Figure 3:**
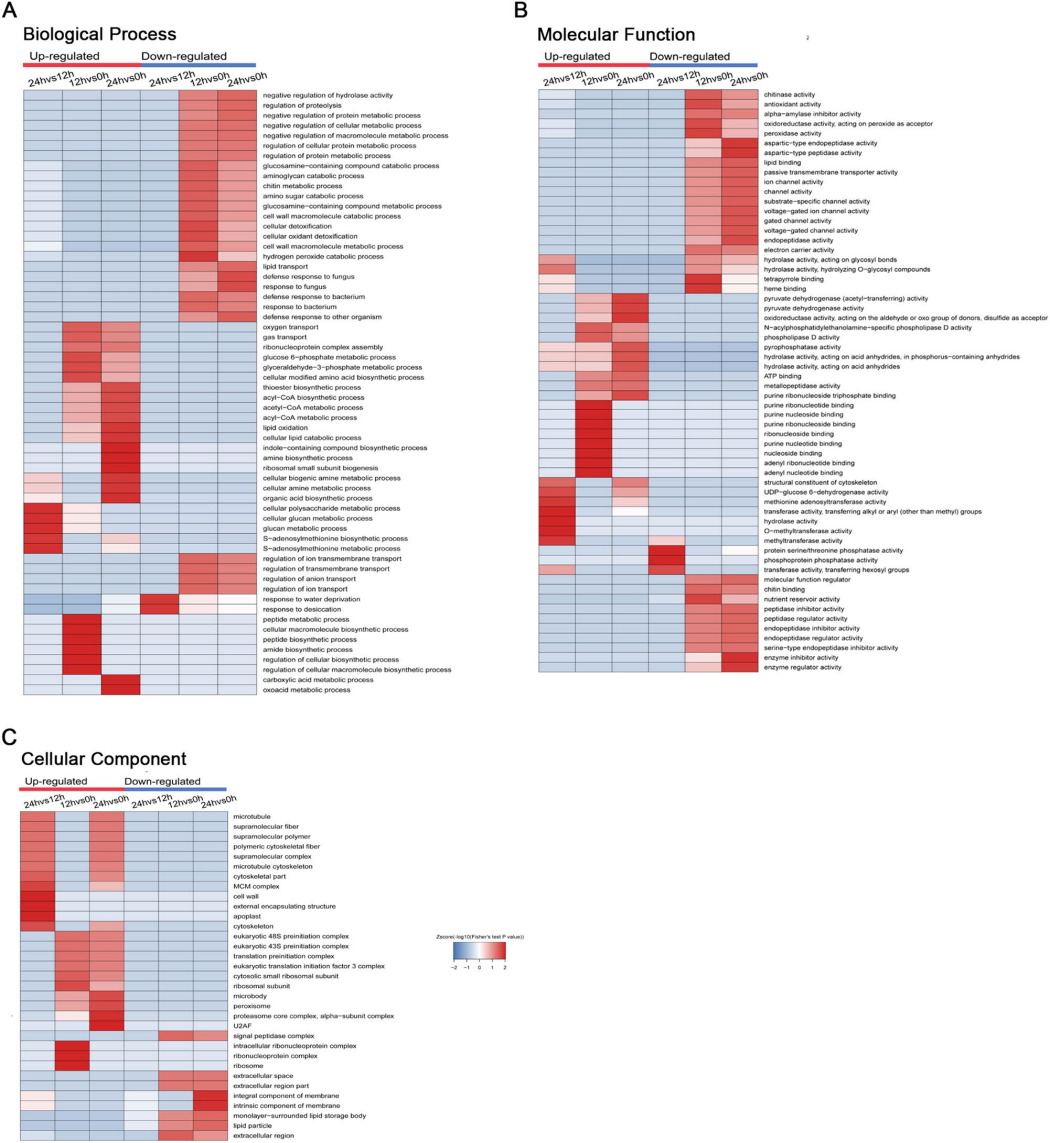
GO-based enrichment analysis of whole-cell proteins in terms of biological process (**A**), molecular function (**B**) and cell component (**C**).

To better elucidate the nature of the DEPs at different wheat seed germination periods, GO enrichment based clustering analysis was performed in the category of biological processes, cellular components and molecular functions (Fig. 3). In the biological process, we found many material metabolism and biosynthesis related processes were significantly enriched in 12 HAI and 24 HAI treatments induced up-regulated protein groups, such as glucose 6-phosphate metabolic process, glyceraldehyde-3-phosphate metabolic process, gas and oxygen transport, acyl-CoA biosynthetic and metabolic processes, peptide biosynthesis/metabolic processes and regulation of cellular macromolecule biosynthesis, which is consistent with a previous report that germination was a complex process including many events, such as proteolysis, synthesis of macromolecules, and respiration (Fig. 3A)^15^. Meanwhile, our data also showed that more glucose metabolism-related processes were enriched in 12 HAI induced up-regulated proteins while lipid metabolism-related processes were more enriched in 24 HAI treatment contributed up-regulated proteins, suggesting the process of glucose metabolism might occur earlier than lipid metabolism in wheat seed germination progress (Fig. 3A). The dramatically enriched thioester biosynthesis process, amine biosynthesis process and indole-containing compound biosynthesis process in the 24 HAI induced up-regulated proteins imply secondary metabolism may be another important event in wheat seed germination (Fig. 3A). Moreover, in the 12 HAI and 24 HAI resulted down regulated proteins, several negative regulation related processes were markedly enriched including negative regulation of hydrolase activity, protein, cellular and macromolecule metabolic process, which further revealed the dynamic and active material metabolism and biosynthesis of various biomolecules (Fig. 3A). In the analysis of down-regulated proteins, apart from a fore mentioned negative regulation related process, multiple cell wall related processes including glucosamine-containing compound catabolic/metabolic process, amino glycan catabolic process, chitin metabolic process, amino sugar catabolic process and cell wall macromolecule catabolic/metabolic process were markedly enriched in 12 HAI and 24 HAI stages (Fig. 3A). Similar phenomenon was also observed in defence regulation related processes (Fig. 3A).

Corresponding to the down-regulated cell wall and defence related proteins in biological process analysis, we gained several significantly enriched cell wall and deference or stress response related items in molecular function category (Fig. 3B), such as chitinase activity, antioxidant activity, peroxidase activity and chitin binding. Interestingly, numerous channels related activities were markedly enriched in the 12 HAI and 24 HAI resulted down-regulated proteins (Fig. 3B). As to the up-regulated proteins, as many as 8 ribonucleotide or nucleoside binding related items were dramatically enriched to 12 HAI caused up-regulated proteins, which imply mRNA translation and protein expression related activities has been activated at relatively early stage of seed germination (Fig. 3B). Consistently, the notably enriched translation related protein complex, translation factors and ribosomal related components in 12 HAI treatment activated up-regulated proteins in the cellular component analysis (Fig. 3C). Besides, compared with 0 HAI, 24 HAI resulted up-regulated proteins were prominently enriched to plenty cytoskeleton related components, indicating that cell skeleton regulation and remoulding was an important event in relatively later stage of seed germination (Fig. 3C).

#### KEGG pathway enrichment and protein domain analysis

**Figure 4:**
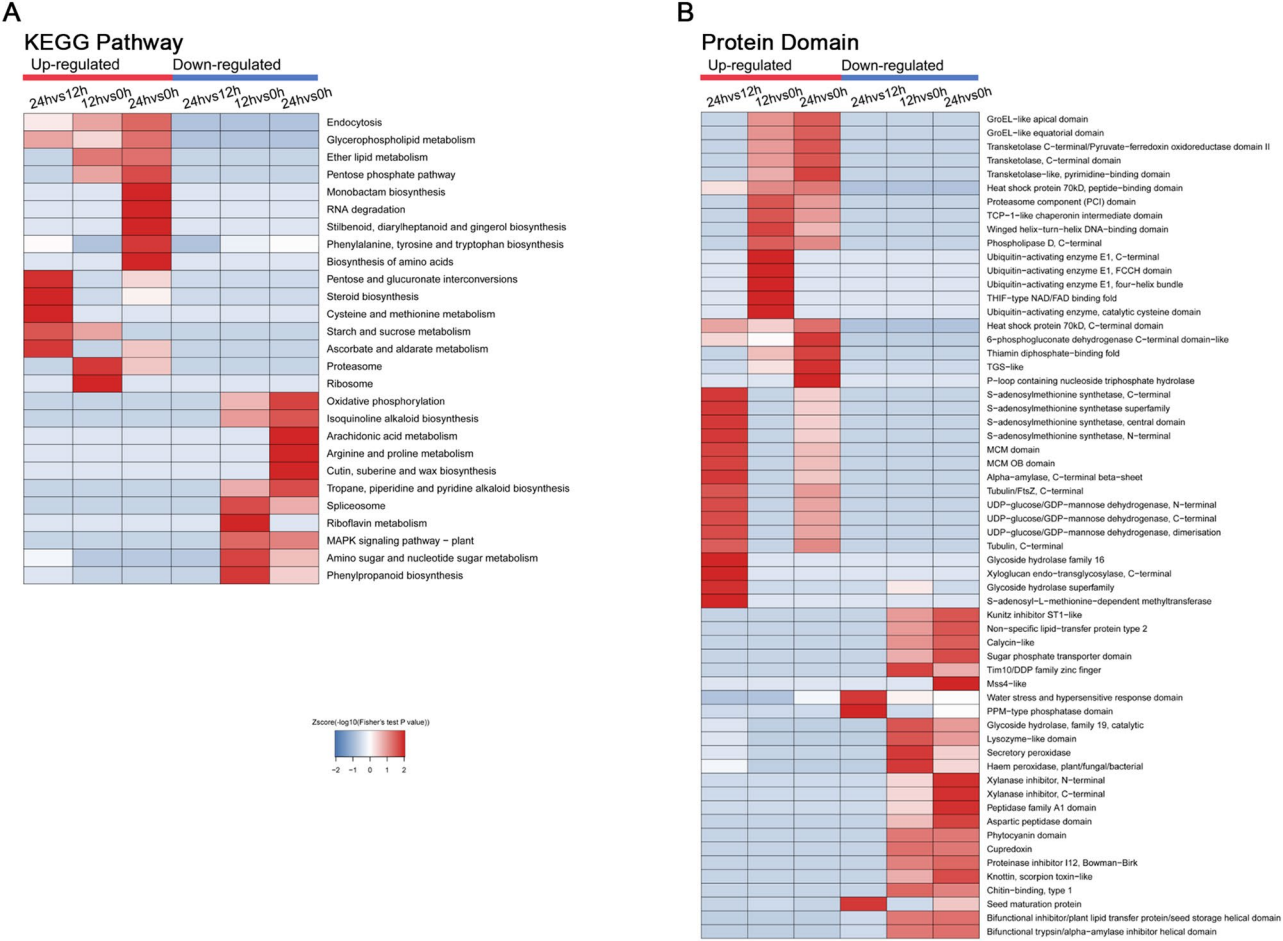
KEGG pathway (**A**) and protein domain analysis (**B**) of whole-cell protein.

To investigate the DEP involved pathways at different germination stages, KEGG pathway enrichment based clustering analysis was implemented (Fig. 4A). In the 12 HAI induced up-regulated proteins, proteasome and ribosome, whose function were definitely opposite, were drastically enriched. Compared with 12 HAI, diverse material metabolism pathways, especially some secondary metabolism related pathway were dramatically enriched, signifying more complex metabolism pathways and complicated biological events participated in later stage germination of wheat (Fig. 4A). The involved metabolites included glycerophospholipid, ether lipid, pentose phosphate, monobactam, RNA, stilbenoid, diarylheptanoid and gingerol, several amino acids, steroid, starch and sucrose, ascorbate and aldarate (Fig. 4A). Han et al*.* reported that large-scale starch mobilization might occur at the late stage of germination^16^. Our results also indicated that proteins involved in starch and sucrose metabolism were more abundant at 12 and 24 HAI through KEGG enrichment analysis (Fig. 4A).

Protein domain analysis showed that ubiquitin-activating enzyme and THIF-type NAD/FAD binding and folding proteins were up-regulated at 12 HAI, while heat shock protein 70 KD and 6-phosphogluconate dehydrogenase C-terminal domain-like were abundant at 24 HAI (Fig. 4B). Interestingly, glycoside hydrolase family 16 and glycoside hydrolase superfamily-related proteins were significantly up-regulated at 24 HAI *versus* 12 HAI. These results implied that starch hydrolysis was more active at 24 HAI, which was the late stage of seed germination. Similar results were also found in rice and barley^10,17^. We concluded that starch mobilization became more and more intense during the germination process, and provided nutriments for subsequent seedling growth.

Concluding the enrichment-based cluster analysis of the DEPs at different seed germination stages, we infer carbohydrate metabolism and energy production and proteins metabolism are vigorous biological activities in wheat seed germination stage, especially at relatively early stage. More biological metabolites including secondary metabolites are involved in seed germination relatively later stage compared with early stage. Cell structure regulation including cell skeleton and cell wall remoulding is also important in wheat seed germination.

### Proteome wide analysis of lysine acetylation in germinated wheat seeds

**Figure 5:**
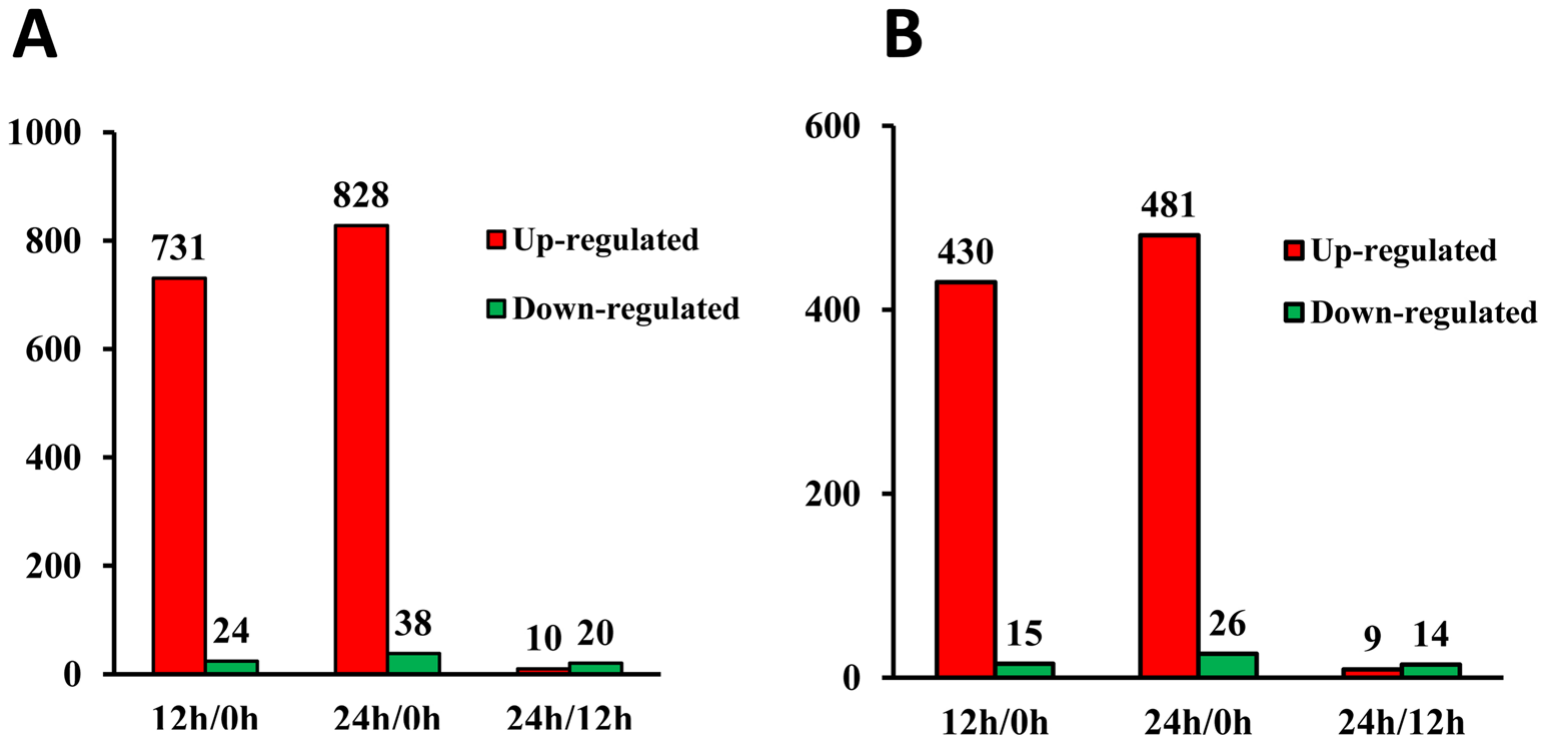
The differentially acetylated sites (**A**) and acetylated proteins (**B**) of germinating wheat seed.

In total, 1,458 acetylated sites in 791 proteins were identified, among which 1,301 sites in 722 proteins were accurately quantified (Supplementary Table S3). With threshold change fold > 1.5 and *P* < 0.05, differentially acetylated sites (DAS) and differentially acetylated proteins (DAP) among three germination stages in wheat embryos were summarized in Fig. 5 and Supplementary Table S4. Obviously, more acetylated sites and proteins showed increased acetylation level at 12 HAI (731 sites in 430 proteins) and 24 HAI (828 sites in 481 proteins) compared to 0 HAI while less down-regulated acetylation sites and proteins were identified in 12 HAI vs 0 HAI group (24 sites in 15 proteins) and 24 HAI vs 0 HAI group (38 sites in 26 proteins), implying protein acetylation may be an active event in wheat seed germination. In addition, compared with 12 HAI vs 0 HAI group, more up-regulated acetylation sites and proteins were detected in 24 HAI vs 0 HAI group, suggesting that the level of lysine acetylation was gradually increased during seed germination process.

**Figure 6:**
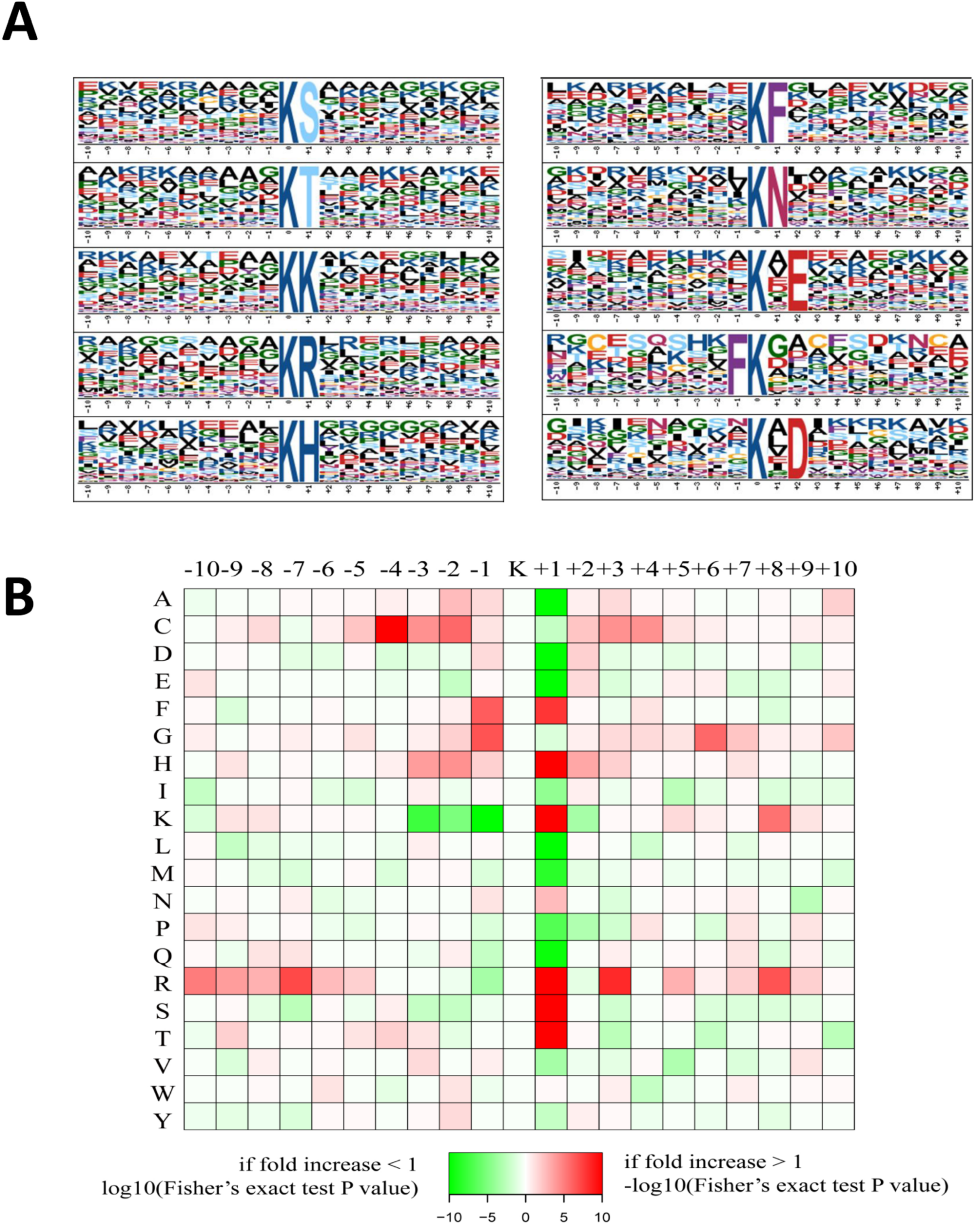
Properties of lysine-acetylated peptides. (**A**) Acetylation sequence motifs for ± 10 amino acids around the lysine acetylation sites. (**B**) Heat map of the amino acid compositions of the acetylated sites.

To understand the properties of acetylation sites and the features of the acetylated peptides in wheat embryo, motif analysis was conducted with Motif-X program which calculated the likelihood of amino acids being over- or under-represented in the positions surrounding the acetylation site. As shown in Fig. 6A, ten consensus sequence motifs were enriched, including K^ac^S, K^ac^T, K^ac^K, K^ac^R, K^ac^H, K^ac^F, K^ac^N, K^ac^*E, FK^ac^ and K^ac^*D (K^ac^ indicates the acetylated lysine, and * indicates a random amino acid residue). Some of these motifs, such as K^ac^H, K^ac^F and FK^ac^, were also found in wheat leaves^11^, and K^ac^K was found in grains of 20 days after flowering^13^. In maize, K^ac^K, K^ac^R and K^ac^F were also identified as the conserved motifs^18^. These overlapped motifs suggested proteins with particular amino acid residues surrounding lysine were more likely to be modified with acetyl groups in crops.

A heat map was generated to show the enrichment or depletion of specific amino acids neighbouring the Kac sites (Fig. 6B). Consistent with the identified conserved motif K^ac^F and FK^ac^, we found phenylalanine (F) was highly represented in the − 1 and + 1 position near acetylation sites, indicating it is a welcomed amino acid either upstream or downstream of the acetylayed sites. In addition, histidine (H), arginine (R), serine (S) and threonine (T) were significantly overrepresented in the + 1 position, corresponding to the identified four motifs in motif analysis. We also noticed that positively charged amino acid arginine (R) was significantly over-represented at multiple positions surrounding acetylation sites, both the downstream and the upstream of acetylated sites. Lysine (K) was greatly enriched in the + 1 position but was greatly depleted in the upstream (− 3 to − 1 position) of acetylated sites, which was an interesting phenomenon. Other amino acids appeared with relatively higher frequency around acetylated sites including cystine (C) and glycine (G). What’s noticeable was that several amino acids including alanine (A), aspartic acid (D), glutamate (E), leucine (L), methionine (M), proline (P), and glutamic acid (Q) were excluded from the + 1 position.

Based on the motif result and heat map, we inferred that sequences with F, R, H, S, T near K residues probably be preferred targets of lysine acetyltransferases in embryos of germinated wheat seeds.

### Enrichment-based cluster analysis of the DAPs

#### GO enrichment based cluster analysis

**Figure 7:**
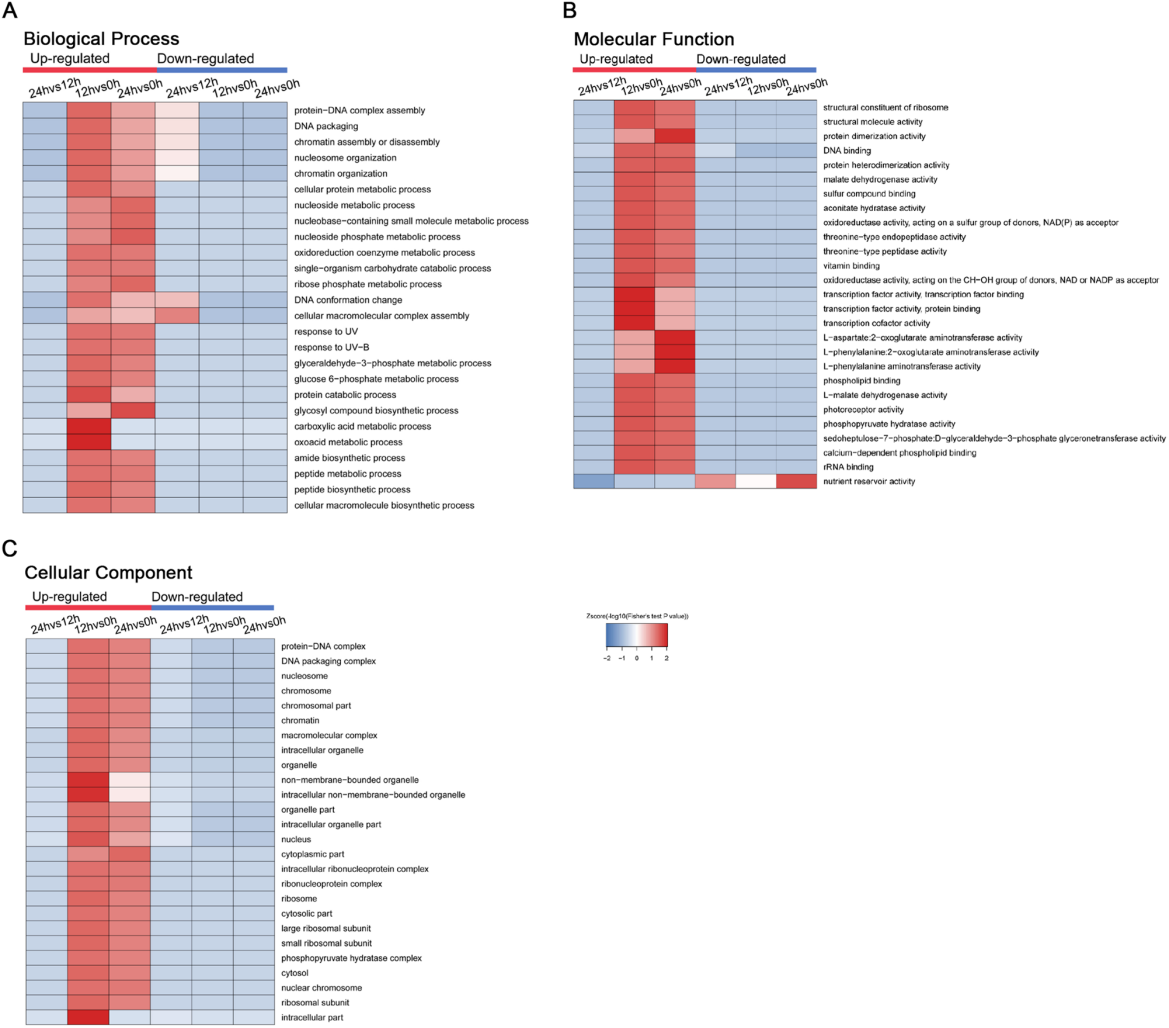
GO-based enrichment analysis of Lysine Acetylation in terms of biological process (**A**), molecular function (**B**) and cell component (**C**).

To better understand the possible roles of lysine acetylation in germinating wheat seed, GO enrichment analysis was performed (Fig. 7). Compared with 0 HAI, acetylated proteins showed similar enrichment, and the largest proportion of Kac proteins was up-regulated at 12 and 24 HAI based on the GO analysis. According to biological process enrichment, various metabolic processes, translation and biosynthetic processes were greatly enriched for acetylated proteins (Fig. 7A). During the germination of cereal seeds, the energy demand is fulfilled mainly by glycolysis. The embryo of most germinated seeds exhibits a basic pattern of oxygen consumption. Briefly, seeds imbibe a considerable amount of oxygen first and then enter a lag period until oxygen levels are sufficient^19^. When the radicles break through the episperm, germination is complete, and oxygen consumption increases sharply. Consistent with these reports, we observed that proteins associated with carboxylic acid and oxoacid metabolic processes were highly abundant at 12 HAI, suggesting that lysine acetylation was associated with energy metabolism (Fig. 7A).

Regarding molecular function analysis, proteins with binding activity and catalytic activity were both enriched at the 12 and 24 HAI stages (Fig. 7B). In addition, proteins related to transcription factor activity were enriched in the 12 HAI embryos. Previous studies showed that many transcription factors were found in the embryos of germinated rice seeds^20^. Proteins related to amino transferase activity were more enriched at 24 HAI due to *de* novo protein synthesis, which was necessary for germination to occur^21^. All these data indicated that acetylation was very important for protein synthesis.

Accordingly, in the analysis of cellular component enrichment among proteins with up-regulated acetylated sites, cytosolic-, cytoplasmic-, and cytosol-related categories were greatly enriched (Fig. 7C). The glycolytic pathway was mainly performed in the cytosol, and our results were consistent. Meanwhile, the nucleosome, DNA packaging complex, protein-DNA complex and ribosome were also observed in enrichment proteins. These results suggested that acetylation might play an important role in transcriptional regulation, protein synthesis and DNA repair during seed germination in wheat.

#### KEGG pathway enrichment and protein domain analysis

**Figure 8:**
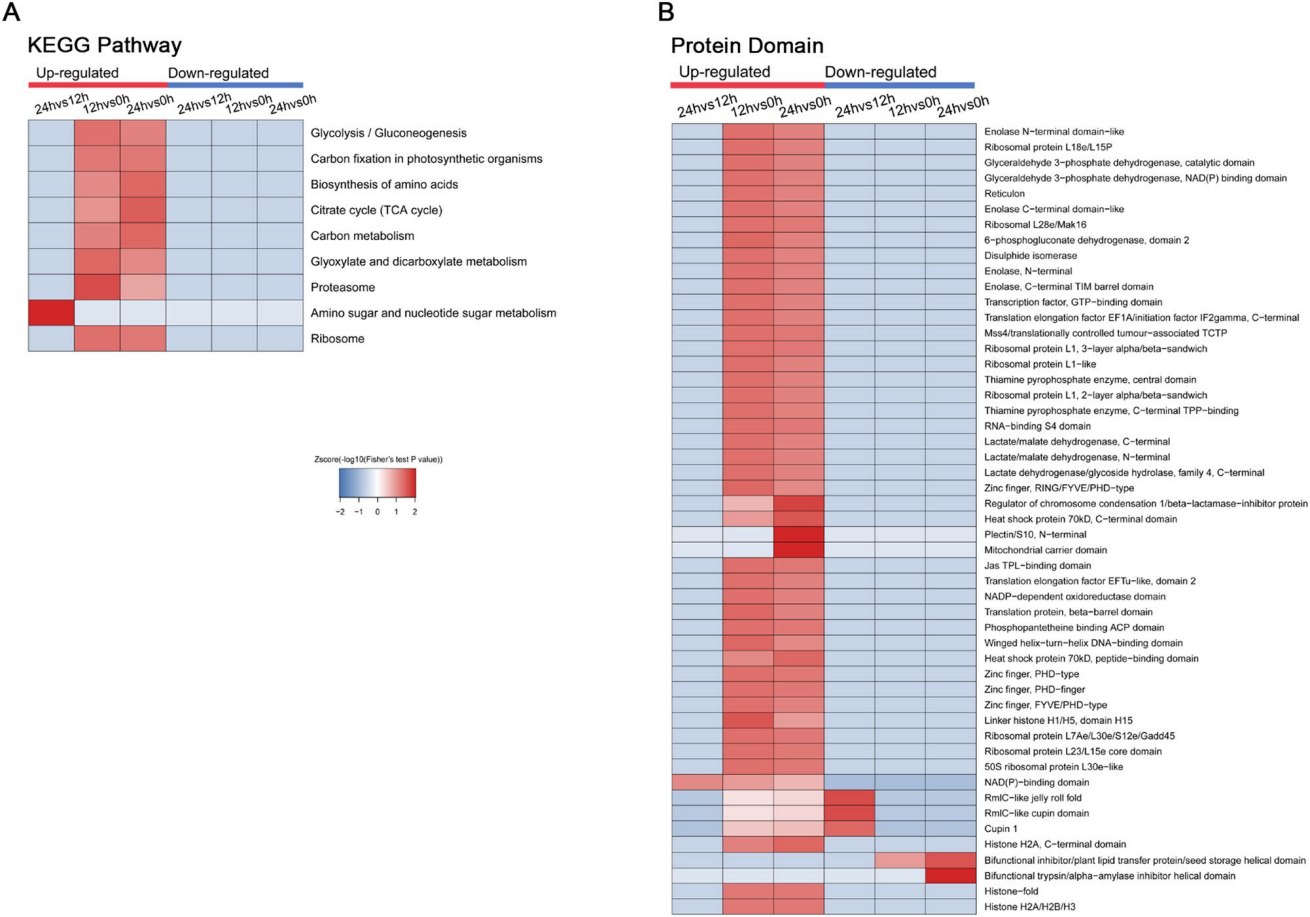
KEGG pathway (**A**) and protein domain analysis (**B**) of lysine acetylation.

To better understand the general function of these acetylated proteins in embryos during wheat seed germination, KEGG pathway analysis was performed (Fig. 8A)^22^. The results showed that lysine acetylation was associated with the Ribosome, Glycolysis/Gluconeogenesis and Carbon fixation in photosynthetic organisms pathways in both 12 and 24 HAI samples compared with 0 HAI samples. In addition, Glyoxylate and Dicarboxylate Metabolism and Proteasome pathways were more enriched at 12 HAI. Furthermore, three categories were more enriched for acetylated proteins at 24 HAI, including Biosynthesis of amino acids, Citrate cycle (TCA cycle) and Carbon metabolism. Yang et al*.* found that TCA cycle efficiency was not high in the early germination stages, and energy was supplied mainly by an aerobic respiration due to limited oxygen content^23^. These results suggested acetylated proteins had a broad range of biological functions in the transition from fermentation metabolism to respiratory metabolism during the transition from seed germination.

To discover further possible functions of proteins affected by lysine acetylation during seed germination, we performed protein domain enrichment in Kac proteins (Fig. 8B). Histones as well as metabolic and stress response proteins were mainly enriched at 12 and 24 HAI. It was notable that the mitochondrial carrier domain family was significantly enriched at 24 HAI given that this family functions to produce energy for the cell through respiration, suggesting that Kac affected different processes in germinating seeds.

### Analysis of histone acetylation in the embryos associated with wheat seeds germination

Histone acetylation is a reversible modification, affecting gene expression as well as the structure and transcriptional competence of chromatin^24^. The acetylation of core histones is typically associated with gene activation, whereas histone deacetylation is associated with gene repression^25^. A previous study revealed that H4K5ac was most evident in the scutellum in embryos from germinating seeds of *Brachypodium distachyon*^26^. However, such information on the precise Kac sites in histones during wheat seed germination was poorly studied. Here, we detected 101 lysine-acetylated peptides matched to differentially expressed histones. In the comparison of histone acetylation among the three stages, more acetylated sites on histones were observed in 12 and 24 HAI (Supplementary Table S5). A total of 84 acetylated sites were common in 12 and 24 HAI among the up-regulated proteins; 7 and 8 acetylated sites were specifically noted in 12 h vs. 0 h and 24 h vs. 0 h, respectively. One acetylated site of H3.2 was common in 12 h vs. 0 h, 24 h vs. 0 h, and 24 h vs 12 h. The different acetylation profiles of the histones between the two stages indicated that acetylation was differentially involved in epigenetic regulation during seed germination.

A recent review demonstrated several histone deacetylases (HDACs) regulated histone deacetylation participated in seed germination^27^. Our study further ascertained the crucial role of histone acetylation in seed germination and expanded the study of histone acetylation in seed at germination stage.

### Analysis of hormone signalling pathways associated with seed germination

Seed germination is the process by which the dried seeds break dormancy until the radicle is observed^10^. Studies have shown that ABA plays an important role in the maintenance of seed dormancy, and ABA concentration can influence the breakage of dormancy in the seeds^28,29^. ABA levels are controlled by two key enzymes: 9-cis-epoxycarotenoid dioxygenase (NCED) and ABA 8′-hydroxylase (CYP707A)^30^. The Cytochrome P450 707A (CYP707A) gene family catabolizes ABA, and the corresponding reduction in ABA levels permits GA synthesis and the events surrounding germination to subsequently begin^15^. In this study, we found that the P450 protein (CYP71C8v2) (Q8S9E7) was acetylated at 12 and 24 HAI, and the acetylated level was higher at 24 HAI than 12 HAI (Supplementary Table S4). Additionally, one of the major negative regulators of ABA signalling pathway, PP2C, was reported to interact with SnRK2s and turn ABA signalling off^31^. In this study, the acetylated level of PP2C was higher at 12 and 24 HAI than 0 HAI, but there was no difference at the protein level among these three stages (Supplementary Table S4).

ABA does not work directly; it functions through the expression of a series of ABA-responsive genes. Previous studies had found that many ABA-responsive genes were highly expressed in mature dry seeds, but the genes related to the elongation of the imbibition of seeds were largely debilitated in *Arabidopsis* and barley^32,33^. The 14–3–3 proteins function as an important element in the ABA signal transduction cascade and could interact with HvABI5 to influence seed germination in barley^32^. This is a key target of the conserved ABA signalling pathway in plants; thus, transcript and protein accumulation, stability and activity are highly regulated by ABA during germination and early seedling growth^34,35^. In this study, the acetylation of 14–3–3 (A0A1D5XQA5) was up-regulated at 12 HAI and 24 HAI, but there was no difference in the ABF/AREB/ABI5 family among the three stages (Supplementary Table S4). In addition to participating in plant hormonal signalling, proteins of the 14–3–3 family also have well-defined functions as regulators of primary metabolism and ion homeostasis in plants^32^. We hypothesized that the up-regulated 14–3–3 proteins played other roles that depart from the plant hormone pathway during wheat seed germination. In addition, acetylation of ABA-inducible ABRC3 proteins was down-regulated at 12 HAI and 24 HAI. All these data implied that ABA levels were not influenced, but ABA-responsive proteins were down-regulated during seed germination.

Seed germination is controlled by the balance of two antagonistic plant hormones, positive GA and negative abscisic acid (ABA)^36^. In contrast to ABA, high GA levels promote seed germination through causing the secretion of hydrolytic enzymes, which weaken the structure of the seed testa^37^. However, in our results, we did not find differentially expressed proteins related to GA biosynthesis or transportation among the three stages. It is likely that GA was synthesized in the scutellum and then diffused to starchy endosperm^38^, but this was not the case in the embryo tissue sampled in this study.

Auxin is a major plant hormone required for plant development and it can be transported from its synthetic site to its functional site^39,40^. SNX1, the first described plant sorting nexin, can help PIN2 recycle or degradation^41,42^. Vacuolar protein sorting (VPS) proteins, the key components of plant retromer complex, can work together with SNX1 in common development pathway^40,43^. In this study, we found that the acetylation of SNX1 (A0A1D5V9E7) and VPS72 (W5I2N4) was up-regulated at 12 HAI and 24 HAI (Supplementary Table S4). These results suggested that plant hormone signalling pathway might be associated with seed germination.

### Stress-related proteins participate in wheat seed germination

Given changes in external environments, wheat seeds may activate a series of mechanisms to respond to biotic and abiotic stresses during germination^44^. We found 28 acetylated sites in 14 stress-related proteins at 12 HAI and 53 acetylated sites in 27 stress-related proteins at 24 HAI had acetylation changes (Supplementary Table S4), indicating that stress-related proteins played important roles in wheat seed germination. Moreover, some previously identified stress related proteins were also identified, such as the 70-kD heat shock protein (HSP70)^45,46^ and late embryogenesis abundant (LEA) proteins^47–49^. We found that the acetylated sites of HSP70 (A0A1D5S806, A0A1D5TAS8, W5DYF8, W5E0J0) (Fig. 5B, Supplementary Table S4) and an LEA homologue (high similarity with At3g53040) (A0A1D6RXD9) was highly acetylated at 12 and 24 HAI (Supplementary Table S4). These proteins were also found acetylation in maize, rice and barely^10,17,18^. Collectively, our results indicated that a wide range of important regulatory pathways were responsive to seed germination.

DnaJ proteins, one of the important stress responsive proteins, can influence several processes in cells through activating the ATP enzyme activity of HSP70^50^. The number of DnaJ proteins varies from species to species. For example, there are 89 DnaJ proteins in *Arabidopsis*^51^, 41 DnaJ proteins in humans^52^ and 22 DnaJ proteins in yeast^53^. Moreover, a few DnaJ genes have been identified in rice, but the function of DnaJ proteins in wheat is barely known. In our study, two acetylated sites of the DnaJ protein homologue was highly acetylated at 12 HAI (A0A1D5YJS6) and 24 HAI (A0A1D5SPU4) respectively. These results suggest that DnaJ proteins may play an important role in wheat seed germination.

### Changes of proteins involved in carbohydrate metabolism during wheat seed germination

Wheat seed germination is a complex process that requires large amounts of energy and nutrition. Because the germinating seed lacks a mineral uptake system and photosynthetic apparatus, the energy must be provided by the seed itself. Starch is the major reserve in mature wheat seed. Upon imbibition, the starch granules are first degraded by α-amylase. Then, the large-branched glucans are catalysed by debranching enzymes to form linear glucans. Subsequently, the linear glucans can be attacked by β-amylase, and the maltose product can be further degraded into glucose by a-glucosidase. In addition, linear glucan can be degraded into shorter glucans and subsequently glucose. Alternatively, α-1,4 glucan phosphorylase catalyses glucan into glucose-1-phosphate. Through the synthesis and degradation of sucrose, glucose can be transferred into glucose phosphate and fructose phosphate. A previous study implied that large-scale starch mobilization might occur during the late germination stage^23^. Our results were consistent with the study. Specifically, α-amylase increased at 24 HAI, but the isoamylase was up-regulated at 12 and 24 HAI. We did not find acetylated site differences on these enzymes during seed germination. These results implied that an increase in enzymes related to starch degradation in embryos of germinated seeds, but the level of their acetylation did not differ.

Consistent with starch degradation, the speed of glycolysis and TCA cycle increased, and these pathways provided the main energy for germination. Many enzymes involved in glycolysis were identified as up-regulated proteins during germination^20,23^. Three key enzymes are involved in the process of glycolysis: hexokinase, phosphor-fructokinase and pyruvate kinase. In our study, one acetylated site of phosphor-fructokinase (W5ARV3) was found at 24 h vs. 0 h, and two acetylated sites of pyruvate kinase (A0A1D5UPJ6) were up-regulated at 12 h vs. 0 h and 24 h vs. 0 h (Supplementary Table S4). Otherwise, five and six acetylated sites of two aldolase enzymes (A0A1D5VD12, A0A1D5VE15) were up-regulated at 12 and 24 HAI, respectively; seven and eight acetylated sites of three glyceraldehyde-3-phosphate dehydrogenase enzymes (A0A1D6ABM1, A0A1D6AJ84, C7C4X1) were up-regulated in 12 and 24 HAI, respectively (Supplementary Table S4). Pyruvate dehydrogenase degrades pyruvic acid into acetyl-CoA under oxygen-rich conditions, which subsequently enters TCA cycle^54^. We found that one acetylated site of one pyruvate dehydrogenase enzyme (A0A1D6S8F1) was up-regulated at 12 and 24 HAI (Supplementary Table S4).

In the late germination stages, the energy is supplied mainly by TCA cycle. He et al*.* found that most TCA cycle-related proteins, such as aconitate hydratase, dihydrolipoyl dehydrogenase 1, and alpha-glucan phosphorylase, were generally up-regulated during wheat seed germination^27^. We also observed that the acetylation levels of citrate synthase (A0A1D5WVN9, A0A1D5ZQF2, W5H1Q1), aconitate hydratase (A0A1D5XL34, A0A1D6AQV8) and dihydrolipoyl dehydrogenase (A0A1D5S7R1, W5A874) were up-regulated by greater than twofold at 12 HAI and 24 HAI (Supplementary Table S4). These data suggested that lysine acetylation played an important role in carbohydrate metabolism during wheat seed germination.

### A putative metabolic pathway of acetylproteins is involved in wheat seed germination processes

**Figure 9:**
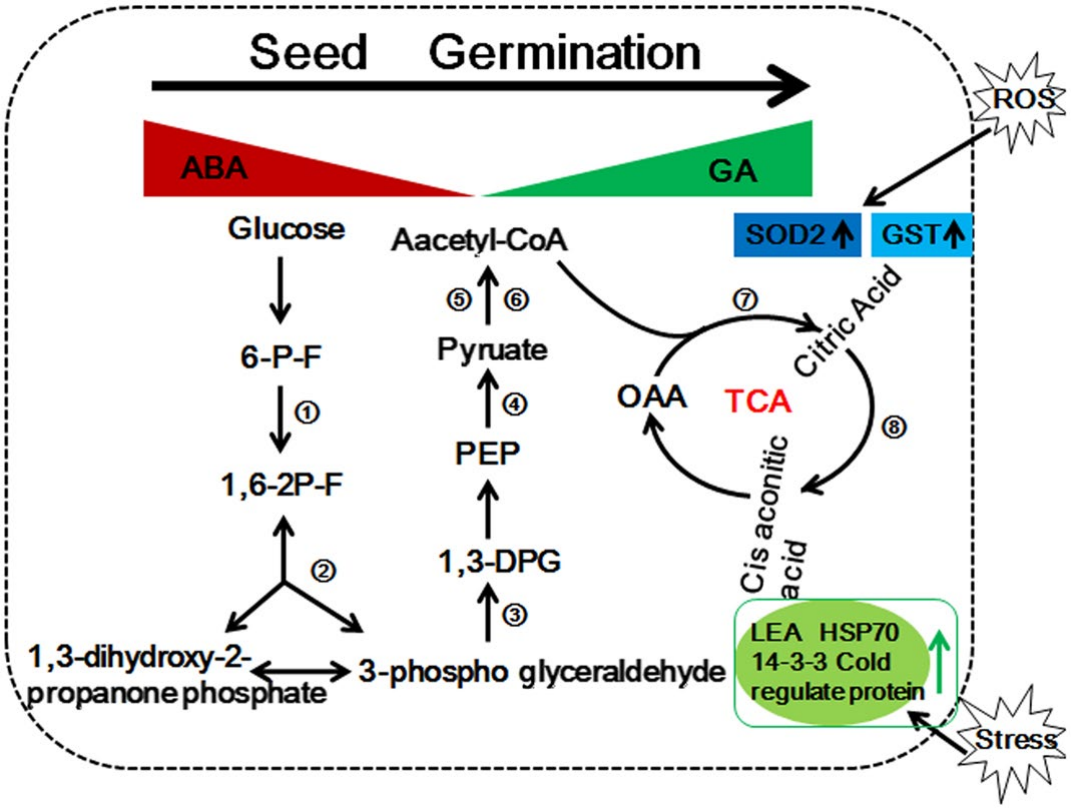
Putative metabolic pathway of acetylated proteins involved in wheat germination processes. Blue indicates antioxidant enzymes; black indicates proteins related to carbohydrate metabolism, and green indicates stress-related proteins.

In this study, we determined the lysine acetylome of germinated wheat seeds at 0, 12 and 24 h after imbibition. Our findings showed that acetylated proteins were associated with various biological functions and are distributed in a number of cellular compartments of embryos during germination. As shown in Fig. 9, at the beginning of germination, ABA was catabolized by the Cytochrome P450 707A (CYP707A) (Q8S9E7) gene family, and the low level of ABA permits GA synthesis. GA then induces the synthesis of β-amylase in the aleurone layer, which secretes hydrolysis enzymes to the endosperm. Subsequently, wheat seeds consume a significant amount of oxygen and then enter a lag period. In this stage, glycolysis is the dominant pathway for energy supply. The key enzymes phospho-fructokinase (W5ARV3), pyruvate kinase (A0A1D5UPJ6), aldolase (A0A1D5VD12, A0A1D5VE15), and glyceraldehyde-3-phosphate dehydrogenase (A0A1D6ABM1, A0A1D6AJ84, C7C4X1) were up-regulated at 12 HAI or 24 HAI. We identified one pyruvate dehydrogenase (A0A1D6S8F1) and dihydrolipoyl dehydrogenase (A0A1D5S7R1, W5A874) that could catalyse pyruvic acid to acetyl-CoA, and both were up-regulated at 12 and 24 HAI. When the radicles break through the episperm, the oxygen consumption increases sharply, and the TCA cycle becomes the main source of respiration. The acetylation levels of citrate synthase (A0A1D5WVN9, A0A1D5ZQF2, W5H1Q1) and aconitate hydratase (A0A1D5XL34, A0A1D6AQV8) were up-regulated at 12 HAI and 24 HAI. Among them, one acetylated site of A0A1D5WVN9 (K266) was up-regulated by greater than twofold in 24 HAI vs. 12 HAI, and the K822 of A0A1D6AQV8 was significantly acetylated at 24 HAI, suggesting that TCA cycle mainly occurred in the late stage of seed germination.

Some stress-related proteins and ROS-scavenging proteins were activated during wheat seed germination^23^. On imbibition, ROS levels increased, which could result in oxidative stress. The antioxidant enzymes that could scavenge ROS efficiently, such as glutathione S-transferase (A0A1D5VDS2) and superoxide dismutase (SOD2), were significantly acetylated at 12 HAI or 24 HAI. Meanwhile, the acetylated level of some other stress-related proteins, such as LEA (A0A1D6RXD9), HSP70 (A0A1D5S806, A0A1D5TAS8, W5DYF8, and W5E0J0), 14–3-3 protein (A0A1D5XQA5) and cold regulated protein (Q8H0B8) were also up-regulated at 12 HAI or 24 HAI. These important proteins cooperate and provide a sound basis for regulation of seed germination and subsequent seedling growth.

## Conclusions

In conclusion, this study provides a global and comparative analysis of proteome and acetylome regulation during seed germination and offers further insights into the dynamics of individual acetylated sites. Our results revealed that K^ac^H, K^ac^F, FK^ac^ and K^ac^K were conserved in wheat. We also revealed that histone acetylation is differentially involved in epigenetic regulation during seed germination. Abscisic acid (ABA) and auxin signalling pathways played a major role in seed germination of wheat. Stress related proteins were also found with acetylation changes, some of which have been reported to be associated with seed germination and others are unknown in plants. Several enzymes that play important roles in glycolysis, the TCA cycle and ROS-scavenging proteins were also acetylated. The provided data set may serve as an important resource for functional analysis of lysine acetylation in germinated wheat seeds. Thus, the changes unveiled by omics in this study provide new insights into the molecular mechanisms of seed germination.

## Materials and methods

### Plant materials and seed germination

The seeds of common wheat variety (*Triticum aestivum* L.) Qing Mai 6 (QM6), which was released by Qi Lin (Qingdao Agricultural University) in 2007 and displays normal growth under 0.3% salt concentration, were washed with distilled water for five times and then imbibed with distilled water in a dark growth chamber at 21 °C. The embryos with three biological replicates (600 mg per replicate) were collected at intervals of 0, 4, 8, 12, 16, 24 and 32 HAI, respectively. Then the embryos were snap-frozen with liquid nitrogen for 1 min and stored at − 80 °C for the proteome or acetylome analysis. Three biological replicates were performed for each germination stage in all the analyses.

Germination efficiency was determined in triplicate. Fifty uniform seeds were selected and washed with distilled water for five times. Seeds were placed in Petri dishes (90 mm in diameter) containing two layers of filter paper and 12 ml of distilled water. Plates were then placed in a 21 °C growth chamber in the dark. Seeds were considered to be germinated when radicle protrusion was visible. Seed germination was scored regularly for 0–32 h.

### Protein extraction

Proteins were extracted from common wheat seeds following previous report with some modification^10^. Briefly, embryos were grinded in liquid nitrogen and homogenized in lysis buffer (8 M urea, 1% Triton-100, 10 mM dithiothreitol (DTT), and 1% Protease Inhibitor Cocktail. The remaining debris were depleted through centrifugation at 15 000 g for 15 min at 4 °C. Then the supernatant was precipitated with ice-cold acetone for more than 4 h at − 20 °C and then centrifuged at 15 000 g for 15 min at 4 °C. The obtained protein was washed with cold acetone for three times and stored at − 80 °C for further use.

### Trypsin digestion

Firstly, the protein was dissolved in buffer (8 M urea, 100 mM NH_4_HCO_3_, pH 8.0). Then the protein solution was reduced with 5 mM DTT for 30 min at 56 °C following alkylating with 11 mM iodoacetamide for 15 min at room temperature in darkness. After dilution with 100 mM TEAB to reduce urea concentration to less than 2 M, a two-step trypsin digestion was carried out according to Zhang et al*.*^11^. After digestion, peptide was desalted by Strata X C18 SPE column (Phenomenex) and vacuum-dried.

### TMT labelling

The dried peptides were labelled with a TMT kit (Thermo Fisher Scientific) under the manufacturer’s instruction^55^. Briefly, peptides were resuspended in 50 mM HEPES, and TMT reagent was dissolved in acetonitrile. Then each TMT reagent was mixed with each peptide sample and incubated at room temperature for 2 h and pooled, desalted and vacuum dried.

### HPLC fractionation and affinity enrichment

After labelling, the peptides were fractionated into fractions by high pH reverse-phase HPLC using Thermo Betasil C18 column with mobile buffer A (98% H_2_O and 2% acetonitrile with 10 mM ammonium formate, pH 10) and mobile buffer B (2% H_2_O and 98% acetonitrile with 10 mM ammonium formate). The LC gradient initiated at 2% and increased to 60% buffer B for 80 min to generate 80 fractions. Then the 80 fractions were combined into 18 fractions for the global proteome analysis and 8 fractions for lysine acetylome analysis^56^.

For affinity enrichment, the fractions of peptide were incubated with pre-washed pan anti-acetyl lysine antibody beads (Cell Signaling Technology, Danvers, USA) in NETN buffer (100 mM NaCl, 1 mM EDTA, 50 mM Tris–HCl, 0.5% NP-40, pH 8.0) at 4 °C overnight with gentle shaking. After washing four times with NETN buffer and twice with double distilled water, the lysine acetylation peptides bound to the agarose beads were eluted with 0.1% trifluoroacetic acid^57^. Finally, the eluted fractions were combined and vacuum-dried for further use.

### LC–MS/MS analysis

The dried peptides were first dissolved in 0.1% formic acid (FA) and separated using versed-phase analytical column (15 cm length, 75 μm i.d.) on an EASY-nLC 1,000 UPLC system^10^. Then, the peptides were subjected to NSI source followed by tandem mass spectrometry (MS/MS) in Q Exactive TM Plus (Thermo Scientific) coupled online to the UPLC system. Detection of intact peptides were performed in the Orbitrap at a resolution of 70,000 (m/z 200) with Normalized Collision Energy (NCE) setting of 28. To scan MS, the m/z range was set from 350 to 1,800. A data-dependent procedure that alternated between one MS scan followed by 20 MS/MS scans was applied for the top 20 precursor ions above a threshold ion count of 3E4 in the MS survey scan with 15.0 s dynamic exclusion. Automatic gain control (AGC) was used to prevent overfilling of the Orbitrap; 2E5 ions were accumulated for generation of MS/MS spectra. The voltage for electrospray analysis was set at 2.0 kV^54^. The fixed first mass was set at 100 m/z for TMT quantification. LC–MS/MS analysis was performed blindly by PMT Biolab Hangzhou (Hangzhou, China).

### Database search

The obtained MS/MS data of acetylation peptides were processed using MaxQuant with integrated Andromeda search engine (v.1.5.2.8). The tandem mass spectra were searched against UniProt Triticum database (146,090 sequences, released March, 2015) concatenated with reverse decoy database^58^. The trypsin/P was specified as cleavage enzyme, allowing up to four missing cleavages. Mass error was set to 20 ppm in first search and 5 ppm in main search for precursor ions and 0.02 Da for fragment ions. Carbamido methylation on cysteine was specified as fixed modification, whereas oxidation on methionine, acetylation on Lysine and acetylation on protein N-terminal were specified as variable modifications. Reporter ion was set as 10 plex-TMT for quantification. False discovery rate thresholds for protein, peptide and modification site were specified at 1% and minimum score for modified peptides was set > 40.

### Bioinformatics analyses

The proteome of Gene Ontology (GO) annotation was derived from the UniProt-GOA database (https://www.ebi.ac.uk/GOA/)^58^. Kyoto Encyclopedia of Genes and Genomes (KEGG) database and InterProScan were used to annotate protein pathway and protein domains respectively. The GO, pathway and domain with a corrected *P*-value < 0.05 were considered significant. Soft motif-X was used for motif analysis of lysine acetylation sites^58^. The enrichment-based clustering analysis was performed with R-package following the previous descripted procedure^45^.

### Statistical method

SPSS 17.0 was used for data process and the measured data were indicated as mean ± SEM. Comparisons between groups were tested by One -Way ANOVA analysis and statistical difference was set with *P* < 0.05.

## Supplementary information

Supplementary Information.

Supplementary Table S1.

Supplementary Table S2.

Supplementary Table S3.

Supplementary Table S4.

Supplementary Table S5.
