# Supplementary material for: Proteome and lysine acetylome analysis reveals insights into the molecular mechanism of seed germination in wheat
Source: Sci Rep. 2020 Aug 10;10:13454. doi: 10.1038/s41598-020-70230-8 (PMC7418024; doi:10.1038/s41598-020-70230-8)
Supplement: Supplementary file 1 — Supplementary Information. [file 41598_2020_70230_MOESM1_ESM.docx]

**Proteome and lysine acetylome analysis reveals**

**insights into the molecular mechanism of seed germination in wheat**

**Weiwei Guo^1#^, Liping Han^1#^, Ximei Li^1^, Huifang Wang^1^, Ping Mu^1^, Qi Lin^1^, Qingchang Liu^1,2^, Yumei Zhang^1*^**

^1^ Shandong Provincial Key Laboratory of Dryland Farming Technology/Shandong Engineering Research Center of Germplasm Innovation and Utilization of Salt-tolerant Crops/College of Agronomy, Qingdao Agricultural University, Qingdao Shandong 266109, China

^2^Laboratory of Crop Heterosis and Utilization, Ministry of Education, China Agricultural University, Beijing 100193, China

^#^These authors contributed equally to this work

^*^Corresponding author: Yumei Zhang, Tel: +86-532-86080446, Email: [zhangcui2003@163.com](mailto:zhangcui2003@163.com)

**Supplementary material**

**Supplementary Table S1.** Annotation of the total proteins in germinating wheat seed embryos.

**Supplementary Table S2.** The differentially expressed proteins (DEPs) among three selected HAI intervals.

**Supplementary Table S3.** The acetylated sites of proteins in germinating wheat seed embryos.

**Supplementary Table S4.** The differentially acetylated sites (DAS) and differentially acetylated proteins (DAP) among three germination stages in wheat embryos.

**Supplementary Table S5.** Differentially expressed proteins of histone acetylation.
